# *País de gordos/país de muertos*: Obesity, death and nation in biomedical and forensic genetics in Mexico

**DOI:** 10.1177/0306312715608449

**Published:** 2015-12

**Authors:** Vivette García-Deister, Carlos López-Beltrán

**Affiliations:** Departamento de Biología Evolutiva, Facultad de Ciencias, Universidad Nacional Autónoma de México (UNAM), Mexico City, México; Instituto de Investigaciones Filosóficas, Universidad Nacional Autónoma de México (UNAM), Mexico City, México

**Keywords:** biopower, citizenship, co-production, death, forensic genetics, genomic medicine, obesity, publics

## Abstract

This article provides a comparison between genomic medicine and forensic genetics in Mexico, in light of recent depictions of the nation as a ‘*país de gordos*’ (country of the fat) and a ‘*país de muertos*’ (country of the dead). We examine the continuities and ruptures in the public image of genetics in these two areas of attention, health and security, focusing especially on how the relevant publics of genetic science are assembled in each case. Publics of biomedical and forensic genetics are assembled through processes of recruitment and interpellation, in ways that modulate current theorizations of co-production. The comparison also provides a vista onto discussions regarding the involvement of genetics in regimes of governance and citizenship and about the relationship between the state and biopower in a context of perceived health crisis and war-like violence.

Two statistics have dramatically shaped the perception of the Mexican nation over the past decade: the first is the number of people who suffer from obesity, and the second is the count of murder victims. According to the latest report issued by the Food and Agriculture Organization (FAO, 2013), Mexico is now the world’s most obese nation. In Mexico, 72 percent of women and 67 percent of men are overweight or obese (Instituto Nacional de Estadística y Geografía (INEGI), 2010). This condition is linked to a number of diseases, most notably type 2 diabetes, which is prevalent in over 8 percent of the adult population.

As for the number of murder victims per year, the estimates range from the government’s official figure of 9000 (Procuraduría General de la República) to 16,000 (Comisión Nacional de los Derechos Humanos) or more. In part because they are linked to the government’s war on drugs, these numbers are disputed, and counts released by official institutions are frequently criticized as being too low, challenged in public debates and contrasted with different sources.^1^

During the 6 years of President Felipe Calderón’s term of office (2006–2012), the annual rate of diabetes-related deaths mounted so much that, in the end, deaths resulting from violence and diabetes reached similar numbers (Althaus, 2013). In the social imaginary of public opinion, the country’s two leading causes of death (diabetes and homicide) were encoded in two different depictions of Mexico. On one hand, it is a country officially described as a ‘*país de gordos*’ (country of the fat) and, on the other, it is unofficially depicted as a ‘*país de muertos*’ (country of the dead). Genetics has been brought to bear on both images. Headlines such as ‘Mexicans are fat because of a gene’^2^ or ‘Mexicans susceptible to diabetes due to genetic factors’^3^ are frequent, while reporting of drug-related murders increasingly includes references to the DNA identification of the human remains.^4^

It is in this setting and time frame that two programmes of ‘national priority’ were launched: one, implemented by Mexico’s National Institute for Genomic Medicine (INMEGEN), is a genomic medicine project, while the other, led by the Attorney General’s Office (PGR), calls for a national forensic genetics. The first is a public health initiative with the objective of developing ‘a more individualized, predictive and preventive medicine based on a person’s genetic factors of protection or risk’, especially to type 2 diabetes (INMEGEN, 2009), while the second, according to the official announcement, is intended ‘to strengthen the database of genetic profiles of the national public security information system in order to constitute it as a tool for law enforcement’ (Morales, 2012). Both initiatives are instantiations of a classic kind of science–government coalition, which has been identified by STS investigators as products of ‘negotiations between researchers and other political actors [that] infuse scientific knowledge with the assumptions and worldviews of both scientists and their sponsors’ (Cozzens and Woodhouse, 1995: 536). Transfer of these assumptions into professional practice and scientific policy contributes to their shaping of the public image of science.

Comparison of genomic medicine and forensic genetics allows us to examine how the biopower of genetics – the role of genetics in providing apparently vital clues to the body and its bio-cultural substance, and the deployment of these clues for the purpose of governing the nation and its population – is progressively acquiring a privileged status in crucial nodes that link citizens, society and state. The comparison also opens up a vista onto debates about how genomics figures in regimes of governance and citizenship: as both a resource via which citizens can make claims on the state for justice and accountability in a situation of violence, impunity and corruption, and a resource that the state can use to recruit its citizens into a project of hope, progress and well-being (and profit).

This article will examine continuities and ruptures in the public image of genetics that operates in these two areas of attention (health and security), focusing especially on *which* are the ‘relevant publics’ of genetic science in each case, and *how* they are ‘assembled’ practically (Hinterberger, 2012). We also evaluate the ways in which the public deployment of genomic medicine and forensic genetics creates ‘spaces of encounter’ for genetic discourse (Warner, 2002).

We will draw on multi-sited fieldwork carried out in Mexico City from 2005 to 2013.^5^ Our attention to the parallels between the two programmes was brought about by the fact that our observations of biomedical genetics in the public space at this time were constantly interacting with the strident claims around forensic genetics, as the spectacle of the war against and among the *narcos* (drug-traffickers) became a major social reality. In response, we became observers of both sets of issues. For this reason, we refer to material gathered from the following mixed sources: (1) ethnography of national and international conferences in medical genomics and ethnography of protests and demonstrations regarding violent death and disappearance; (2) semi-structured interviews with professionals in areas related to genomic medicine (researchers, spokespeople) and violence and death (advocacy groups, public servants); and (3) news media coverage of public health campaigns regarding diabetes/obesity, and government press releases and local and international assessments of violence and the use of forensic genetics in Mexico. Our findings suggest that there are distinctive trajectories and features of ‘public-ization’ (Hayden, 2003, 2004) at work in biomedical and forensic genetics in Mexico. A crucial contrast we establish is that of people’s experience of blood donation in each setting, which takes place in a context of either confidence or mistrust towards the state and, we argue, is a form of public engagement with genetic science.

## Assembling the publics of biomedical and forensic genetics

National forensic DNA databases and national genetic profiles developed for biomedical purposes are both political and economic investments of considerable magnitude, especially relative to Mexico’s government expenditure, and they have taken on an iconic status as a measure of a nation’s ability to deal with contemporary health and security issues. Mexico’s programmes follow ones that have been in place for a number of years in countries like the United Kingdom, the United States, Iceland and Germany, which have been subject to consideration by the literature on biopolitics as well as science, technology and society (STS) research (Koenig et al., 2008; Krimsky and Simoncelli, 2011; Krimsky and Sloan, 2011; Tutton, 2004).^6^

DNA collection from donors is crucial to large-scale biomedical research projects aimed at mapping genomes and identifying the genes implicated in diseases of public health concern. Hinterberger (2012) has argued that donors may constitute both the relevant populations under study and the publics whose deliberations are, at least theoretically, consulted as genome science is underwritten and used by the state. This distinction accurately points to the various strategies that have been employed by governmental power to identify, recruit and standardize various groups into nationally defined populations, for example, ethno-racially as ‘Mexican diabetics’ (Montoya, 2011) or as ‘Mexican mestizos’ (López-Beltrán et al., 2014).^7^ This distinction also recognizes that scientists, together with other political actors, are interested in ‘assembling publics’ that are ‘vested with particular forms of agency’ (Hinterberger, 2012: 530; Latour, 2005). The promissory and speculative nature of genomic science as an economic activity (Fortun, 2008) is premised upon a future consumer pool (O’Riordan, 2013) that will pay, directly or indirectly (through taxes, private insurance, out of pocket, etc.), for risk management devices. The identification of specific genetic variants considered to predispose certain individuals to disease is central to these assemblages insofar as they become a tool for delineating the future consumer market.

At least eight genetic variants of relevance to Mexicans have been identified. For example, in 2010, the ABC-A1 gene variant R230C (rs9282541) was associated with obesity and type 2 diabetes in Mexican mestizos and described as a ‘functional variant exclusive to Native American and descent populations’ (Acuña-Alonso et al., 2010: abstract). By 2012, six other gene variants^8^ had been linked to type 2 diabetes in Mexican mestizos (Gamboa-Meléndez et al., 2012). A recent study involving data from more than 8000 DNA samples of Mexicans and other Latin Americans identified SLC16A11 as a novel candidate gene for type 2 diabetes in these populations (SIGMA Type 2 Diabetes Consortium et al., 2014). It has been found that this variant, while common in individuals of Native American or Amerindian descent, is rare in European and African samples. Substantially funded by the Slim Initiative in Genomic Medicine (which combines the public–private investigative efforts of INMEGEN and the BROAD Institute of MIT and Harvard), the objective of the Consortium is to develop pharmacological treatment based on prediction of risk to developing the disease. This goal remains elusive, however. There is an on-going international debate ‘within diabetes sciences about the cost-benefit of researching the genetics of complex diseases in light of the methodological complexity and immense uncertainty that the findings will result in any beneficial outcomes’ (Montoya, 2011: 44). In some headquarters, genomics is considered to be ‘only a marginal contribution to public health’.^9^ A recent study that analyses the gene–lifestyle interaction of diabetes shows that ‘information on common genetic variants associated with the risk of type 2 diabetes offers little improvement for risk prediction over and above established type 2 diabetes risk factors’, including lifestyle (Langenberg et al., 2014).

The rhetoric of large-scale genomics projects fences off these sober and sceptical accounts of the predictive powers of genome scanning, replicating a pattern similar to what O’Riordan (2013) found in studying direct-to-consumer genomics. The discourse on genomic medicine by its advocates tends to emphasize a generalized sense of its promise and empowerment. In a document titled ‘Genomic medicine as a strategic instrument for Mexico’s development’, INMEGEN director Jiménez-Sánchez (2003) strung together the key arguments of a discourse he would repeat almost without variation throughout his directorship, lasting until November 2010. First, genomic medicine was poised to become one of the great tools of preventive health care, since it held out the possibility of identifying individuals genetically vulnerable to common diseases before they could contract them. Second, genomic medicine would give rise to new treatment strategies through the invention and use of drugs targeting the genomic structure and epidemiological characteristics of the Mexican population; these would be more effective, less toxic and less prone to side effects. Third, genomic medicine would not only serve as an economic multiplier by boosting scientific and technological development of new products and services, but it would also help reduce public health expenditure by more efficiently treating the diseases that mostly affect the general population. These arguments addressed an intersecting set of interests: from those of the Ministry of Health to those of Congress and from those of the pharmaceutical entrepreneur to those of the general population. By repeating the same mantra in diverse media, the notion of genomics percolated down to the general population. INMEGEN even sponsored the production of comic books aimed at a younger public and inserts for business periodicals.

**Figure 1.**
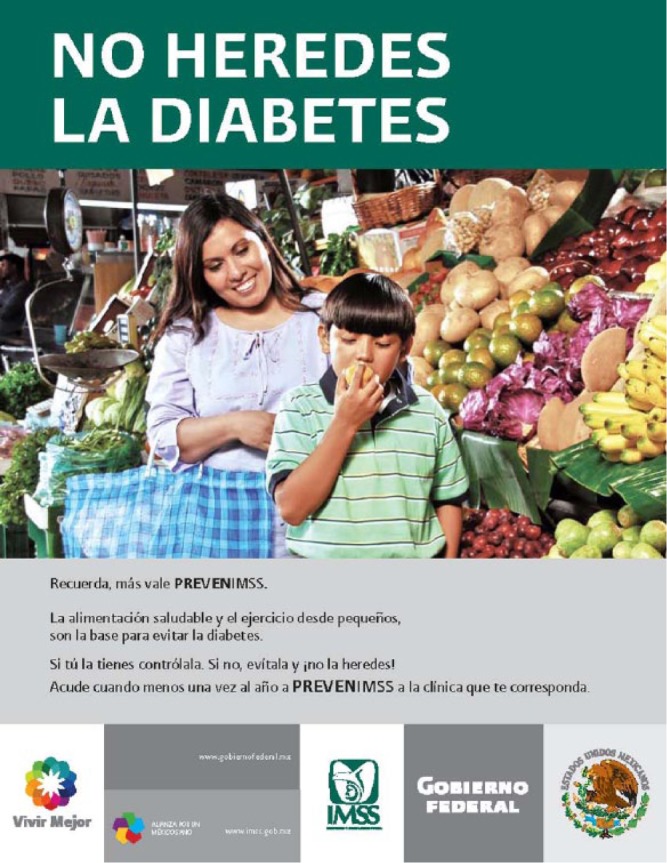
Government campaign concerning diabetes, which ran during the years 2008–2009. Text: Do not inherit diabetes. A healthy diet and exercise, from childhood, are the basis for prevention of diabetes. If you have it, control it. If you do not, avoid it and do not inherit it!^11^

Four years after INMEGEN was created, discourse about genetics and the heritability of diabetes had gained a place in public opinion. Figure 1 is an example of a message that the Ministry of Health issued in a public health campaign concerning diabetes. ‘Do not inherit diabetes’, reads the legend above a photograph of a mother and her child in a market setting surrounded by fruits and vegetables. By reference to heredity, the ad raises the possibility that there are genetic components of the disease, without explicitly mentioning its cause or treatment, thus inviting a mediated ‘genomic’ reading. At the same time, in the Mexican imaginary, physical and bodily dispositions are passed down from one generation to another through processes that are not only biological. That is, diabetes occupies a place within complex and diversified narratives of heredity, which is why the visual suggestion to consume healthy, local foods is meant to drive the hybrid bio-cultural message home. The example illustrates how publics are assembled from audiences that exceed the ad’s specific text by interacting with and postulating other forms of discourse.^10^ The articulation of these texts and interactions through time is what Warner (2002) has called the ‘scene of address’.

During the presidency of Calderón, the government spent over 2000 million pesos (US$155 million) on publicity in the health sector, within which campaigns to combat obesity and diabetes were very conspicuous. Compared with the previous administration of Vicente Fox, Felipe Calderón left office with a 35 percent increase in deaths related to diabetes (totalling almost 500,000) and more than 200 percent increase in the cost of this treatment, which translated into a state of crisis for the health system (Calvillo, 2012).

INMEGEN’s initial promise of a predictive, preventive and personalized medicine has been postponed, since ‘achieving it has been more difficult than expected’.^12^ All the same, the good news, as the Institute insists, is that the ‘opportunity for Mexico to … develop new markets and opportunities for business development’ (Jiménez-Sánchez et al., 2010: 550) has been duly seized. An ethno-racial national niche market is taking form through a process that could be described using Hacking’s (1986) idea of ‘making up people’, that is, reshaping the way in which persons are understood, engaged and handled by actors such as policy makers and providers of public health services. An array of terms has emerged in the literature to describe the assemblages that make up the publics/niche markets of genomic science: ‘perpetual patients’ (Pálsson, 2007), ‘genomic patients’ (López-Beltrán, 2011) or ‘prospective patients’ (O’Riordan, 2013). All are ‘inferred or imagined publics’ (Anderson, 1983; McNeil and Haran, 2013), rather than corporeal subjects capable of assembling themselves and forming support groups (such as those described by Epstein, 2008). These terms refer to biopolitical and statistical entities shaped by ‘the speculative futures of potential drug markets [and] public health concerns’ (Montoya, 2007: 94). In Mexico, they are a projected percentage of the population who, by the fact that they carry a particular assortment of genetic variants, are more vulnerable to diabetes and/or obesity than the rest of the population and should thus, by inference, be subject at some point to special, joint intervention by state and private healthcare programmes (we say more about INMEGEN’s public relations strategy and its effects on assembling the publics of genomic medicine in the next section).

The publics of forensic genetics, by contrast, are much more humanly recognizable as the kinds of affected people who protest, march, use social networks and write bill initiatives. They also make up a large portion of the live, consenting donors of the samples from which forensic DNA repositories are generated (complementing those that are collected from cadaveric remains). Mexico’s national database, which to date contains approximately 15,000 genetic profiles,^13^ has yet to be properly organized and constituted, as does a general regulatory framework for DNA databases.^14^ Officially, this project began as the outcome of an agreement reached during the national conference for law enforcement in the year 2008, when a new system of coordination between federal and local offices was launched. By November 2011, the attorney general’s office had implemented – after laborious negotiation with the Federal Bureau of Investigation (FBI) and the US embassy in Mexico – the FBI’s Combined DNA Index System (CODIS) software. Today, 20 out of 32 federal entities in Mexico have a genetics laboratory in their local prosecutor’s office, but even by the most optimistic estimates the number of unidentified bodies far exceeds the number of successful DNA-based identifications, which fluctuates between 200 and 500, depending on the source. In February 2013, Mexico’s State Department affirmed that the number of disappeared persons during the Calderón administration was 26,121. According to Human Rights Watch (2013), this places Mexico as the country with the largest number of disappeared persons in Latin America’s contemporary history.

Madres y Familiares de Desaparecidos (Mothers and Families of the Disappeared), Familiares de Víctimas de la Guerra Contra el Narcotráfico (Families of Victims of Mexico’s War on Drugs) and Fuerzas Unidas por Nuestros Desaparecidos en Nuevo León (United Forces for Our Disappeared in Nuevo León, FUNDENL) are only three of many advocacy groups that have assembled to put pressure on the authorities to provide real security and to resolve thousands of unresolved cases. As the news about the possibility of securing more certain identities of bodies with forensic genetics becomes publicly available (in part aided by the so-called CSI effect^15^), genetic testing becomes a key discursive element in these demands.

Twenty-five-year-old Brenda Damaris González Solís disappeared from the scene of a car crash in July 2011, in the northern state of Nuevo León. In 2012, Brenda’s family received 166 ‘fragments’ of what was allegedly her body inside a plastic bag. The prosecutor of Nuevo León advised Damaris’ mother not to open the bag and referred to a DNA test that confirmed the body’s identity. But the family never saw a laboratory report or heard a medical examiner’s interpretation. On 29 June 2013, *#PruebagenéticaNL* (#GenetictestNL) became a trending topic on Twitter Mexico. ‘What if they are not her remains?’ was the header on the picture of Damaris posted by FUNDENL, who demanded a second DNA test in order to confirm the identity of the remains provided.

Like other civil organizations addressing similar cases of uncertain identification, FUNDENL brings together affected groups and legal advisers, which in practice creates one particular kind of public for forensic genetics (which we refer to more broadly as advocacy groups). The Coordinator of Human Rights and Citizen Security for FUNDAR, a research and analysis centre, puts the use of DNA technologies within the framework of the Inter-American Commission on Human Rights’ principle that families have a right to know the fate of their loved ones, otherwise known as the ‘right to truth’:

I believe that forensic genetics can help to exercise this right to the truth. I have heard of cases in which people say, ‘These remains that they delivered to me do not belong to my daughter’. In this feeling of doubt that lingers in the victim’s parents there is violence, and it leaves their right to the truth unfulfilled. Some scientific certainty is required, and DNA can provide it. (Human Rights Coordinator at FUNDAR, interviewed by García-Deister, 16 August 2012)

But DNA’s configuration as an instrument of truth has other functions besides that of providing scientific certainty, which further complicates the ‘forensic imaginary’ (Williams, 2010) of advocacy groups. For Ana Lorena Delgadillo, a human rights advocate and long-time collaborator with the Argentine Forensic Anthropology Team, the high degree of technical knowledge required to fully understand the scope and limitations of forensic genetics, together with the poor treatment that families of victims receive from government authorities, leads to two different consequences for the affected groups. Either DNA matching becomes ‘an act of faith for the families’, or precisely because they have been ‘victimized, mocked, refused justice and worn down’ by the institutions in charge of genetic profiling, the families of victims ‘no longer believe in the DNA test’, even after receiving the results (Delgadillo, interviewed by García-Deister, 9 November 2012). This situation is exemplified with the case of Damaris, for whom a second, more ‘truthful’ DNA test is being demanded. Here, the lack of trust is in the law enforcement agency that conducts DNA tests rather than the tests themselves. With the help of FUNDENL, Juana Solís Barrios and Juan Antonio González Vázquez (parents of Damaris) obtained a judge’s authorization to exhume the fragments they had received in 2012 and were pressured to bury in the municipal cemetery, in order to put in motion a second genetic test. Once the authorization was released, Letty Hidalgo, founder and director of FUNDENL, brought the case to Franco Mora from the Peruvian Forensic Anthropology Team during a workshop on forensic search and identification strategies. Simultaneously, Nuestra Aparente Rendición liaised with Gobernanza Forense Ciudadana (GFC) to allocate the funds to perform the DNA test with a private firm in the United States. The disinterment, which was jointly conducted by Peruvian and Mexican Forensic Anthropology Teams,^16^ took place in 10 September 2014. The DNA test results that confirmed Damaris’ identity were released in February 2015.^17^

Thus far, we have shown that publics of genomic medicine are recruited at a ‘scene of address’ (Warner, 2002, cf. O’Riordan, 2013) that emphasizes norms of healthy living and situates the promise of genomics in the development of more efficient medical treatment, with positive consequences for the nation. A second scene of address, the more concrete and terrifying situation of victimization and violent acts, motivates the assemblage of publics of forensic genetics. At the second scene, the power of DNA technologies to produce certain human identification is appreciated. However, the methods of collecting and storing evidence are questioned because the state’s security organisms are not trusted with the chain of evidence in their control. Far removed from the first scene of address, from the progressive impetus with which promoters of genomic medicine have framed research programmes for Mexican audiences, the second scene of address nonetheless solicits political, legislative and media attention. In the next section, we will look more closely at the mechanisms through which discourse about the efficacy of human genetics creates spaces for oriented uses and research projects in genomics.

## Spaces of encounter with biomedical and forensic genetics

By the time INMEGEN was created, massive public engagement activities had become one of the marks of Big Science. Jiménez-Sánchez understood the value of these activities and was familiar with bioscience’s most successful enterprise in terms of public dialogue, the Human Genome Project (HGP), which devoted 3–5 percent of its resources to the ethical, legal and social implications of the venture. With Johns Hopkins colleagues David Valle and Barton Childs, Jiménez-Sánchez proposed a functional classification of disease genes within the human genome, which was published along with the HGP’s first draft in *Nature* (Jiménez-Sánchez et al., 2001). INMEGEN integrated public engagement into its structure and budget in three ways: by deliberately modelling its ethical, legal and social implications unit on the HGP’s; by rhetorically leaning on the prestigious, branded international HapMap Project; and by devoting a large amount of money and human resources to an outreach and education department that produced and distributed public relations and scientific materials. During the years 2005–2009, its main object of ‘public-ization’ (Hayden, 2003, 2004) was its Mexican Genome Diversity Project (also known as the ‘Map of the Mexican genome’). INMEGEN’s combination of public relations and science was on display when its first scientific article (Silva-Zolezzi et al., 2009) was ceremoniously delivered into the hands of President Felipe Calderón. The scientific paper had been preceded by 5 years of ‘preliminary results’^18^ (López-Beltrán and Vergara-Silva, 2013) intensively communicated by INMEGEN through mass media. The promise of a ‘predictive, preventive and personalized medicine’ became the sound bite at the centre of its publicity campaign, one in which politics, science and marketing were treated as interlocking facets of the whole (García-Deister, 2014). In this way, the object of its investigation (the Mexican population at large) was transformed into its audience.

The publics of genomic medicine thus came spectrally into being in virtue of being constantly addressed (Warner, 2002). This attribute has been theorized in terms of the ‘co-production’ of natural and social orders, in the context of the biosciences and their publics (Jasanoff, 2004; McNeil and Haran, 2013). Such a view highlights a concurrence of natural-social orderings, but it also suggests simultaneity at the scenes of address. Our examination of the publics of genomic medicine, by contrast, points to a definite temporal displacement in this co-production. The discursive practices of INMEGEN indeed aim at establishing concurrence between genomic medicine and its recipients, but the idea of genomic medicine precedes engagement with the actual patient of genomic medicine. The public that receives the targeted messages is simultaneously reacting and reshaping itself to conform to the expected medical future. The effects of these practices are similar to those achieved by what Cody (2009, 2011) calls ‘regimes of circulation’: ‘cultivated habits of animating texts, enabling the movement of discourse along predicable social trajectories’ (Cody, 2011: 43). This theorization is attentive to the various space–time registers of genetic discourse and to the dynamics of public encounters: the publics of genomic medicine have been imagined, but the actual recipients of genomic medicine – the future genomic patients (López-Beltrán, 2011) and the corresponding new breed of healthcare practitioners (Harvey, 2011) – are still in the making.

Warner’s (2002) idea that the circulation of discourse promotes further encounters for discourse is particularly pertinent to our examination of forensic genetics. A sharp rise in violence over the past few years has created an increasing awareness of the lack of forensic laboratories and experts, which in turn has led to the creation of new training programmes in national universities, responses to a crisis of confidence in the ability of the state to perform its elementary legitimating task of securing order within its boundaries. The awareness of the lack of forensic tools is part and parcel of the awareness of the deteriorated state of all of Mexico’s policing structures. While it may be needed, the turn to technical fixes can also be read as part of the ruling elite’s attempt to re-structure the scene of address when that scene seems to implicate the state in massive weakness (due to corruption, lack of resources, lack of concern for victims, etc.). Strengthening the national database of genetic profiles has been regarded as a state priority since 2012 and allegedly constitutes part of the primary state-based response to the crisis. The organized reaction of affected publics, those who challenge the forensic and legal work that the authorities deliver, has intensified as well. Demands for accountability also populate the scene, further amplifying the weight given to the discourse of forensic genetics and making its reading even more dynamic and political.

For Ana Lorena Delgadillo, in a context of structural violence and concomitant mistrust, there is an ‘inseparable union between the technique and the person or institution’ who uses it; thus, the best way to preserve the benefits of DNA technologies requires ‘the existence of independent commissions’ (Delgadillo, interviewed by García-Deister, 9 November 2012). Moreover, she argues that the success of linking science and justice depends on the interactions between experts and affected groups:

The expert has to sit down and talk with the family about what he is doing and explain it in the appropriate language so that it is not an act of faith … but most importantly, because the family deserves a dignified treatment. And this means having clear and objective information about how their blood sample will be used to carry out a comparison, which is not currently done [in Mexico]. (Delgadillo, interviewed by García-Deister, 9 November 2012)

She continues,

Families today are demanding the use of DNA, but independently. There is an awareness of the benefits of DNA but these are not attributed to its use by the attorney general … What would benefit us the most as a country, to improve in terms of justice and human rights, is to have expert services that operate independently from the public prosecutor’s office. (Delgadillo, interviewed by García-Deister, 9 November 2012)

Over the past 14 years, one such expert service, the Argentine Forensic Anthropology Team (EAAF), has liaised with the Mexican government, with varying degrees of success, to implement international recommendations to improve Mexico’s procedures for forensic investigations. In September 2013, Mexico’s Attorney General’s Office, EAAF and a score of Central American non-governmental organizations (NGOs) and civil society associations signed a collaborative agreement to identify the victims of three massive killings of migrants in the northern states of Tamaulipas and Nuevo León (Convenio, 2013). The collaboration surfaced in the midst of implementation of Proyecto Frontera, a regional initiative, involving EAAF; the Mexican state of Chiapas; the governments of El Salvador, Honduras and Guatemala (the most violent countries in the region); and regional NGOs. Proyecto Frontera sought to collect both information on missing migrants and DNA samples of their families in southern Mexico and Central America, to compare with the bodies and remains found in clandestine mass graves across the Mexican territory. Insofar as it evidenced the incapability of the state to manage the emergency (that continues to this day), the project initially faced hostility from the federal government (Turati, 2012). However, after much pressure from international observers, the government ceased its rhetoric of opposition and changed to one of cooperation. This transformation is a patent example of how the encounter between critical discourse circulating at the regional and international levels, on one hand, and official discourse on forensics at the national level, on the other, generates new discursive arrangements.

Both in the biomedical effort to geneticize common diseases and in the organized (official and non-official) attempts to restore dignity to the massacred, discourse about genetics has been immersed in conflictive political situations. We now turn to the different ways in which publics engage with biomedical and forensic genetics.

## Blood donation as public engagement with genetics

‘Blood production is involved in the performance of different types of contract’ (Pfeffer and Laws, 2006: 3014), each of which produces a different kind of engagement with the inputs and outputs of genetic analysis. Medical genetics and forensic genetics provide two cases of donation, importantly differentiated by both motivations for and processes of donation: vials are obtained via venipuncture in the case of medical genetics, whereas blood donation for forensic genetics may involve a finger pinprick and a blood spot card. In both cases, consent forms mediate the dissimilar trajectories of the samples, which are processed and stored differently, and which take up different kinds of space. Experiences of both kinds of donation may evoke kinship and heredity, as each situates the donor in an imaginary relationship with their ancestry or with their next of kin. Genealogical ancestry reconstruction through genomics has become valuable for health risk assessment (but see the first section above) and may be read by blood donors as a prolonged family medical history and as a source of anxiety (López-Beltrán, 2014). However, in forensic genetics, the techno-political specificity of genetic analyses is about a different kind of anxiety linked to kinship. Here, the goal of forensic genetics is to reclaim the disappeared for both the family and the social order: through kinship analysis, the family will either definitely know that one of its members has been killed after the discovery of bodily remains, or will know that it still does not know, if the identification is negative.

In the cases of biomedical and forensic genetics, how their respective publics interact with the black box of genetics depends on how genetic science receives the input of organic samples (typically blood) in order to produce the outputs that feed back into the public sphere, rearticulating links and identities. Publics are always at both ends (input and output) of this process, but are recruited into the ‘machine’ of genetics in varied ways. What we find in these cases are scenarios for engagement in the discovery of disease or death in which genetics becomes a device that can structure the dependencies and outcomes of the interactions. In these scenarios, publics tend not to passively process genetic knowledge but rather react and adapt to it, as they come across the institutional fabric, practices and significance of genetics.

Between 2005 and 2007, blood donors for the Mexican Genome Diversity Project came to INMEGEN’s sampling sites, mainly in universities, across the states of Yucatán, Zacatecas, Sonora, Veracruz, Guerrero, Guanajuato, Tamaulipas, Durango, Oaxaca and Campeche, attracted by the publicity in the media. The centres were deliberately set up so that, in addition to giving blood, the donors were given information about the new technology of genetics, including what it might mean in the therapies of the future. Local TV shows and radio programmes featuring Jiménez-Sánchez and his collaborators called upon university students and other citizens to participate in the development of a public health genomics tailored to the needs and features of the Mexican population (Jiménez-Sánchez, 2009).

Donor recruitment was officially construed as a national ‘health good’ (Petryna, 2009), one in which patriotic citizens should be involved: appeal to solidarity was modulated regionally, for each donation site. This discourse was aimed at ensuring that blood donation was read as a ‘gift relationship’ (Titmuss, 1971), where donors selflessly volunteered their time and their tissues to a national scientific effort. The commitments to making the genetic database publicly available, and to returning to the sampling sites in order to deliver the results, cultivated a politics of reciprocity that further reinforced the Institute’s call for donations. INMEGEN collected each donor’s signature on a consent form, and nurses from the local health system extracted the blood samples. Both INMEGEN’s in-house public relations unit and the mass media duly reported on donation campaigns (García-Deister, 2014).

Although INMEGEN staff explained that their project would not immediately bring about medical applications, blood donors experienced their participation as directly related to health. Before extending their arms for blood withdrawal, some donors cautioned nurses that they ‘had already had breakfast’, worried that food consumption would somehow alter the results of the analyses. This confusion of the purpose of blood donation with the purpose of clinical blood testing might illustrate the success of state-mediated discourse about genomic medicine; certainly, the idea of genomic medicine’s significance in improving Mexicans’ health in the future has been internalized by its publics (García-Deister, 2014).

By contrast, blood donation for forensic genetics is a carefully pondered, intimate act that profoundly affects donors. *El Paso Times* journalist Alejandro Martínez, who has been covering femicide (and militating against it) in the border city of Ciudad Juárez for two decades, comments,

Sometimes families resist donating a sample. These are comments made by mothers of the disappeared young women, and I’ve heard the same at the prosecutor’s office. They tell me that the families are reluctant to donate a sample because it means moving one step closer to admitting the possibility that their relative is deceased. Donating a sample is recognizing the possibility that they will find a dead body. In general, samples are collected at the prosecutor’s office. Knowing the history of the dynamics between civil society and authorities, I have no doubt that they [the Committee of Mothers with Disappeared Daughters] have played an important role in pressuring authorities to be more diligent in the collection of these samples. (Martínez, interviewed by García-Deister, 10 July 2012)

Humanitarian crisis propelled DNA technologies into the national public sphere also in the case of Argentina’s Grandmothers of the Plaza de Mayo. This group liaised with international scientists and organized around genetic identification technologies in order to recover their kidnapped grandchildren, whose parents disappeared during the dirty war in the 1970s (Penchaszadeh, 2011; Smith, 2013; Smith and Wagner, 2007). The work of Smith (2008) has shown that questions of individual identity and notions of family and state accountability are integral to DNA technologies employed for identification.

Precisely because blood donation in cases like these is nothing short of a traumatic experience, a particular ‘recruitmentology’ is at work (Epstein, 2008). More often than not, members of the Committee of Mothers in Ciudad Juárez (and other organizations throughout the Mexican territory) make the first approach to the families who have gone to the prosecutor’s office to report a missing relative and encourage them to donate a blood sample. Activism of this kind is necessary because the burden of jump-starting the genetic identification process falls on the willingness of the families to contribute a sample. The very process of genetic identification requires participation of one and preferably two immediate family members (ideally, parents), in order to extract the reference samples against which DNA obtained from cadaveric remains is matched in the pursuit of a positive identification.

The experience of blood donation for genomic medicine and forensic genetics thus elicits different attitudes towards the expert apparatuses of the state. In the first case, a clinic-like space of donation generates confidence; donors trust the state with their tissue samples, and consent to the downstream processing of their sample, on the understanding that it will contribute to developing a broadly construed national health programme. In the case of forensic genetics, blood donation takes place in a context of distrust. The more concrete and terrifying situation of victimization and structural violence lays bare the holes in the chain of custody of which blood samples would be a part.

## Conclusion

The unexpected simultaneous apparition of two state-supported projects, both involving genetic research, provides a way of sounding out the complex relationship between the state and biopower, in a context of a perceived health crisis and war-like violence. On one hand, the highly publicized project of genomic medicine seems to show the state as a future-oriented rational entity, one that controls and diagnoses the genetic makeup of the national population and prepares the way for future health interventions with profit-making private enterprises, using state-supported data and mechanisms. On the other hand, the advance of forensic genetics brings into focus massive state failure to secure the social and civil order, which is on such a scale that the question has shifted from arresting and trying perpetrators to the question of simply identifying masses of anonymous victims. In the latter case, the publics addressed by the science of genetics are, by and large, those whom the government has failed to protect. Mistrust runs so high that the state must rely on NGOs to finesse the acceptance of sampling procedures. And yet, the success of the genomic medicine project at garnering blood donations demonstrates an underlying pool of trust or at least investment in promises of non-specific revolutionary improvements in the future. Our comparison has shown that the publics of genetic science are not just ‘out there’ pre-formed, to be addressed by the state or to address the state: the publics are recruited and assembled. There is no passivity or docility on their side, but rather differentiated economies of trust and mistrust. The operation of biopower and the enactment of citizenship, which occur through these projects, thus depend on complex processes of interpellation and recruitment. ‘Well-being’ and ‘rights’ accrue to these unstable, partial and, in the case of genomic medicine, largely shadowy networks, rather than to ‘citizens’ or ‘the public’.

## Notes

1. There are important differences in the criteria and sources of information for counting homicides in Mexico. The Attorney General’s Office (PGR) and the Secretario Ejecutivo del Sistema Nacional de Seguridad Pública (SENSP) collect all homicides reported by local police officers and prosecutor’s offices (where homicide is determined by legal, rather than medical criteria); Instituto Nacional de Estadística y Geografía (INEGI) gathers information from the Ministry of Health, which takes into consideration only certificates of death by alleged murder issued by medical examiners. Newspapers, journals and other media have set up ‘executometers’ based on inferences regarding the characteristics of the crime scene. For a critical comment on the uses of executometers, see http://www.animalpolitico.com/blogueros-plata-o-plomo/2013/02/11/y-se-hicieron-bolas-con-los-muertos/#axzz2kdrPld00 (accessed 4 February 2015).

2. http://quo.mx/noticias/2012/08/01/mexicanos-gordos-por-culpa-de-un-gen?tag=diabetes (accessed 4 February 2015).

3. http://www.jornada.unam.mx/2013/10/16/sociedad/043n2soc (accessed 4 February 2015).

4. See, for example, the case of 12 young victims who disappeared from a bar named ‘Heaven’ and whose bodies were later found in a pit: http://internacional.elpais.com/internacional/2013/09/06/actualidad/1378419069_987555.html and http://www.excelsior.com.mx/comunidad/2013/08/25/915370 (both accessed 4 February 2015).

5. Fieldwork carried out by García-Deister involved a 1-year laboratory ethnography at INMEGEN (2010–2011, and close follow-up of scientists’ activities at conferences, both specialized and directed to wider audiences, over a longer period of time (2010–2014) (see Wade et al., 2014, Chapters 3, 6 and 7, where this work is more comprehensively reported). Creation of INMEGEN and its flagship project was the object of analysis of the Seminario de Genómica Crítica del Instituto de Investigaciones Filosóficas de la UNAM (2005–2010) led by López-Beltrán (2011). Both authors also followed and/or participated in public protests regarding violence and death between 2006 and 2012.

6. The DNA collections derived from samples used for recreational and personal genomics have also been examined (Webster 2005, Angrist 2009, 2010, Nordgren and Juengst 2009, McGowan, Fishman and Lambrix 2010).

7. The racial substratum of the mestizo both as an identity figure and as an object of scientific research in Latin America has been described and analysed many times (Stepan, 1991; Wade, 2010). Biomedical and anthropological disciplines have consistently given mestizos ‘a majoritarian vocation’ that considers mestizos to be a unique racial category – ‘product of the fusion of two biologically differentiated and relatively internally homogenous groups: the white race and the indigenous race’ – capable of absorbing others via racial unification (Navarrete-Linares, 2014: 72). Mestizaje is thus seen as the basic racialized fabric with which collective ‘wholes’ of this region’s populations can accommodate the ethnically and biologically diverse ‘parts’ (López-Beltrán and García-Deister, 2013).

8. These are rs13266634 (SLC30A8), rs7923837 (HHEX), rs10811661 (CDKN2A/2B), rs4402960 (IGF2BP2), rs12779790 (CDC123/CAMK1D) and rs2237892 (KCNQ1).

9. Lawrence Gostin, O’Neill Institute for National and Global Health Law at Georgetown University, personal communication with García-Deister, 1 June 2011. According to this specialist, governments should continue to focus on the major (social) determinants of health, which are cheaper and more efficient to deal with, than continue to spend vast amounts of money on state-of-the-art technology that has a much more uncertain payoff.

10. The ambiguity of the message may also be read as indicating an enduring argument about the causality of complex pathologies such as diabetes within the medical field. It also reveals a power struggle within the lobbying business circuits in Mexican politics. For decades, the food and drink consumption habits of Mexican middle and working classes, hugely dominated by junk food and sweetened drinks consortia, have set off alarms. The effort to bring these consortia under strict control through laws and taxation has been aggressively resisted by different means. Among the ‘scientific’ arguments deployed by lobbyists is the claim that only those Mexicans who are genetically predisposed to obesity and/or diabetes should change their eating and drinking habits.

11. Current health campaigns have receded to more conventional communications where the public is encouraged to ‘eat properly’, ‘become active’ and ‘take care’, thus depositing the burden of prevention exclusively on the individual’s lifestyle (Ministry of Health, 2013).

12. Words spoken by Guillermo Soberón, current director general of INMEGEN, at the event ‘First decade of genomic medicine in Mexico’ (22 February 2011) at El Colegio Nacional in Mexico City.

13. The number of genetic profiles has fluctuated over the years, but not in ascending order, as expected. In August 2011, the attorney general spoke of 15,618 profiles, and in June 2014, the deputy attorney general spoke of 14,278 profiles. Fluctuations may be due to culling of the database (e.g. by eliminating duplicate items) and the reorganization of the contents of the database (e.g. data obtained from cadaveric samples or from citizens seeking a disappeared family member may be stored separately). For press coverage of this estimate, see http://www.animalpolitico.com/2014/06/el-banco-genetico-para-identificar-desaparecidos-solo-ha-tenido-2-de-exito/#axzz349iKeeu3; http://www.24-horas.mx/pgr-preve-la-creacion-de-programa-de-genetica-fore; http://www.elindependientedehidalgo.com.mx/2013/01/75779; http://www.razon.com.mx/spip.php?article216502 (all accessed 4 February 2015).

14. Only one of Mexico’s federal entities, the state of Chihuahua, has a law that regulates databases of genetic profiles obtained for forensic purposes; it was issued in April 2009. Rayo del Carmen Orea Ochoa (former head of the genetics laboratory in Mexico City’s Institute for Forensic Sciences) and colleagues published in December 2010 a technical proposal for a more general law initiative (the so-called DNA law) which, according to Orea Ochoa, did not receive any attention from legislators (Orea Ochoa interviewed by García-Deister, 6 November 2012).

15. Here, we follow a broad characterization of the ‘CSI effect’, named for the US crime drama television series *CSI: Crime Scene Investigation*, to signify an increase in the public faith in science to bring about the right outcomes in police investigations, rather than a socio-legal effect on jurors, as discussed by Cole and Diosio-Villa (2007). Our use is close to that of Lynch et al. (2008), who consider it to be ‘indicative of awe and anxiety about the perceived power of scientific evidence, particularly DNA evidence’ (2008: x–xi).

16. See http://epafperu.org/en/pronunciamiento-epaf-emaf/ (accessed 4 February 2015).

17. Personal communication with Alejandro Vélez, NAR editor in chief, 30 August and 15 September 2014. Visit http://nuestraaparenterendicion.com/index.php/nuestra-aparente-rendicion/quienes-somos (accessed 4 February 2015).

18. This is similar to what, in Moody’s (2004) popular examination of the race to sequence the human genome, is termed ‘science by press release’.
